# Editorial Comment: Prevalence, management and outcome of cryptorchidism associated with gastroschisis: A systematic review and meta-analysis

**DOI:** 10.1590/S1677-5538.IBJU.2022.02.02

**Published:** 2022-03-01

**Authors:** Natasha T. Logsdon, Luciano A. Favorito

**Affiliations:** 1 Universidade do Estado do Rio de Janeiro Unidade de Pesquisa Urogenital Rio de Janeiro RJ Brasil Unidade de Pesquisa Urogenital - Universidade do Estado do Rio de Janeiro - Uerj, Rio de Janeiro, RJ, Brasil

Silvia Ceccanti 1, Giuseppe Migliara 2, Corrado De Vito 2, Denis A Cozzi 3

J Pediatr Surg. 2021 Jul 13;S0022-3468(21)00494-2.

## COMMENT

Gastroschisis is a paraumbilical abdominal wall defect associated with protrusion of the abdominal content through a defect which cause is not completely elucidated, but there is evidence of an abnormality in the formation and development of the ventral body wall during embryogenesis, resulting in bowel herniation (1). Patients with gastroschisis are associated with malformations outside the gastrointestinal tract in around 10% of the cases, and with abnormalities related to the gastrointestinal tract in up to 25% of cases (2, 3). The association of gastroschisis and undescended testis probably occurs by mechanical factors rather than prematurity (4).

Undescended testis is associated with abdominal wall defects around 30 to 40% of the cases and in these patients the spontaneous testicular descent occurs in about in 50% of the cases (5). In this interesting systematic review published in Journal of Pediatric Surgery in 2021, the authors confirmed that cryptorchidism in gastroschisis appears to occur more frequently than in the normal population with an unfavorable presentation which is represented by originally intrabdominal or prolapsed testis in the majority of cases. This a very interesting paper to pediatric urology.
